# Physical activity levels during COVID-19 pandemic and its associated factors in patients with Chagas disease

**DOI:** 10.3389/fmed.2024.1411977

**Published:** 2024-08-06

**Authors:** Isis Gabrielli Gomes Xavier, Patrícia Mello Andrade, Rodrigo de Lima Vitor, Tayná Cruz Barros, Luciana Fernandes Portela, Marcelo Teixeira de Holanda, Luiz Henrique Conde Sangenis, Gilberto Marcelo Sperandio da Silva, Flavia Mazzoli-Rocha, Fernanda de Souza Nogueira Sardinha Mendes, Andréa Rodrigues da Costa, Marcel de Souza Borges Quintana, Alejandro Marcel Hasslocher-Moreno, Itauá Leston Araujo, Angela Cristina Verissimo Junqueira, Roberta Olmo Pinheiro, Ingebourg Georg, Vitor Barreto Paravidino, Tatiana Rehder Gonçalves, Roberto Magalhães Saraiva, Mauro Felippe Felix Mediano

**Affiliations:** ^1^Evandro Chagas National Institute of Infectious Disease, Oswaldo Cruz Foundation, Rio de Janeiro, Brazil; ^2^Oswaldo Cruz Institute, Oswaldo Cruz Foundation, Rio de Janeiro, Brazil; ^3^Department of Epidemiology, Institute of Social Medicine Hésio Cordeiro, State University of Rio de Janeiro, Rio de Janeiro, Brazil; ^4^Department of Physical Education and Sports, Naval Academy, Brazilian Navy, Rio de Janeiro, Brazil; ^5^Institute of Public Health Studies, Federal University of Rio de Janeiro, Rio de Janeiro, Brazil; ^6^Department of Research and Education, National Institute of Cardiology, Rio de Janeiro, Brazil

**Keywords:** social isolation, physical activity, Chagas disease, pandemic, COVID-19

## Abstract

**Background:**

A better understanding of the consequences of the Coronavirus Disease 2019 (COVID-19) pandemic on lifestyle of patients with Chagas disease (ChD) is of paramount importance to facilitate the implementation of intervention strategies tailored to this specific population.

**Objective:**

The present study aimed to evaluate the level of physical activity (PA) in Chagas disease (ChD) patients during the Coronavirus Disease 2019 (COVID-19) pandemic and its main associated factors.

**Methods:**

This is a cross-sectional study with 187 patients of both sexes, aged ≥18 years, followed in a national infectious disease center (Rio de Janeiro, Brazil). The level of PA was determined by the International Physical Activity Questionnaire short version and expressed in terms of total volume of physical activity (PA) (MET-minutes per week). Individuals were classified as physically active following the 2020 World Health Organization PA guideline. The exposure variables were age, sex, race, marital status, schooling, income *per capita*, number of rooms per domicile, number of residents per domicile, body mass index, clinical form of ChD, COVID-19 antibodies, comorbidities, self-reported anxiety, self-reported depression, self-reported fear, and self-reported sadness. The association between the exposure variables with total PA (as a continuous variable) was determined using univariate and multivariate linear regression models.

**Results:**

Mean age was 61.1 ± 11.6 years. Most (62%) were women and self-declared their race as mixed (50.8%). The percentage of physically active individuals according to was 52%. The variables independently associated with total PA levels were non-white race (Exp β = 1.39; 95% CI 1.02 to 1.90), dyslipidemia (Exp β = 0.73; 95% CI 0.56 to 0.95) and self-reported depression during quarantine (Exp β = 0.71; 95% CI 0.52 to 0.96).

**Conclusion:**

Non-white race was positively associated with total levels of PA, while dyslipidemia, and self-reported depression during quarantine were negatively associated with total levels of PA. The identification of associated factors can facilitate the development of tailored strategies to increase PA levels ChD patients.

## 1 Introduction

Chagas disease (ChD) is a neglected tropical disease caused by the flagellate protozoan Trypanosoma cruzi (*T. cruzi*) that is endemic in 21 countries in Americas, affecting about 6 to 8 million people worldwide (1). About 70 million people in the Americas are still at a high risk of ChD since they are living in areas of active transmission (2). Moreover, due to migratory movements around the world, ChD has exceeded international borders and can be considered a current global epidemic (1).

In 2020, a new viral infectious disease called Coronavirus Disease 2019 (COVID-19) emerged worldwide, caused by the severe acute respiratory syndrome (SARS-CoV-2 virus) (3, 4). The COVID-19 virus rapidly spread around the world, leading the World Health Organization (WHO) to declare a pandemic state only a few months after the first described cases. COVID-19 is a highly contagious disease that accounted to a high number of infected cases and deaths (5, 6). To mitigate the dissemination of new cases of COVID-19, preventive strategies including social isolation were advocated by many scientific societies around the world (7). While these strategies were fundamental for public health, the inevitable negative consequences of social isolation included the disruption of daily routines, reduced social interaction, mental health challenges, changes in eating patterns, and decreased levels of physical activity (PA) (8–10).

Studies conducted during the COVID-19 pandemic period indicate a decrease in overall PA levels, potentially associated with health impairments (11). In this setting, a better understanding of the consequences of the COVID-19 pandemic on patients with ChD is of paramount importance to facilitate the implementation of intervention strategies to improve health of this specific population. To the best of our knowledge, no previous study has examined the PA levels of patients with ChD during the COVID-19 pandemic period. Therefore, the present study aimed to evaluate the level of PA in patients with ChD during the COVID-19 pandemic and its main associated factors.

## 2 Methods

### 2.1 Study design and population

This is a cross-sectional observational study, carried out from November 2020 to June 2021, including ChD patients confirmed by two simultaneously positive serological tests at the time of study entrance (immunosorbent linked to enzyme assay and indirect immunofluorescence), both sexes, aged ≥18 years. All patients were under regular clinical follow-up at Evandro Chagas National Institute of Infectious Diseases/Oswaldo Cruz Foundation (INI/Fiocruz), a national reference center for treatment and research in tropical and infectious diseases located in Rio de Janeiro, Brazil. At the time of entrance, participants had already been under regular follow-up at INI/Fiocruz for at least 6 months (mean of 16 years). Participants were excluded if they had other infectious diseases at the time of data collection, if they were immunocompromised, or if they had non-Chagasic heart disease.

### 2.2 Sample size

The sample size calculation was based on the study published by Puccinelli et al. (12), which estimated a prevalence of physically active individuals of 76.5% in the Brazilian population during the COVID-19 pandemic. Considering the population of patients with ChD regularly followed at INI/Fiocruz in 2020 (around 900 patients), a 95% confidence interval, 6% accuracy estimate, and increasing the sample size by 10% due to losses and refusals, 175 patients were necessary for the present study.

### 2.3 Ethical considerations

All participants received information about the goals and procedures of the study and agreed to participate by signing an informed consent form. The study was approved on 18/09/2020 by the Research Ethics Committee of the Evandro Chagas National Institute of Infectious Diseases in accordance with resolution 466/2012 of the National Health Council (CAAE: 37026320.2.0000.5262).

### 2.4 Study procedures

Patients were invited to participate during their regular outpatient visits and were submitted to study procedures in two moments. The first moment was performed during patient’s regular medical appointment, in which patients signed the informed consent and performed anthropometric measurements and blood collection. The second moment comprised the application of socioeconomic and clinical questionnaires by telephone, within a period of no more than one month after visit one, and the obtention of clinical information from patient’s medical records. The interviews and questionnaires were conducted in Portuguese, the native language of the study participants. The form is available at: https://osf.io/p9qc8/.

### 2.5 Physical activity

The level of PA was determined by the International Physical Activity Questionnaire (IPAQ) short version, previously adapted and validated for use in the Brazilian population (13). The IPAQ-short consists of 6 questions about the duration and frequency of participation in vigorous, moderate, and walking activities over the previous seven days (14). Energy expenditure is expressed in metabolic equivalent of task (METs) using the compendium of PA (15). The total volume of PA was quantified in MET-minutes per week and was calculated by summing up the total time per week spent in light (3.3 METs), moderate (4.0 METs), and vigorous (8.0 METs) activities (16).

Moreover, individuals were classified as meeting the PA recommendations per week using the criteria determined by the 2020 WHO guideline for PA and sedentary behavior as follows: >150 min of moderate-intensity PA, >75 min of vigorous-intensity PA, or > 150 min of a combination of moderate and vigorous PA (17).

### 2.6 Clinical form of ChD

The classification of the clinical presentation of ChD (indeterminate, cardiac, and digestive) and cardiac stages was performed using information from medical records based on clinical, electrocardiographic, echocardiographic, and digestive examination following the criteria of the 2^nd^ Brazilian Consensus of Chagas Disease (18). To facilitate data analysis and following clinical reasoning, patients with cardiac form of ChD were recategorized as cardiac without heart failure and with cardiac failure.

### 2.7 Comorbidities

Information on comorbidities (systemic arterial hypertension, diabetes mellitus, dyslipidemia, obesity, respiratory diseases, cancer, kidney disease, hepatic and other chronic diseases) was obtained using medical records. Anthropometric measures were obtained during the clinical evaluation. Obesity was determined if body mass index [BMI = weight (kg)/squared height (m^2^)] was ≥30 kg/m^2^. Self-reported data on anxiety, depression, fear, and sadness were collected through direct questions to patients (“During the quarantine you presented anxiety? Did you have depression during quarantine? During the quarantine were you afraid? During quarantine did you feel sad?”).

### 2.8 Socioeconomic data

Information on age, sex, race, marital status, educational level, income, number of rooms per domicile, and number of residents per domicile were collected. Race was self-reported as white, black, mulatto, yellow and indigenous. For data analysis, race was recategorized into white and non-white (black, mixed, yellow, and indigenous). The marital status was self-declared by the participant among the options single, married/stable, divorced, and widowed. Schooling was categorized based on the formal years of study into <9 years, ≥9 to 12 years and ≥ 12 years. Family income was determined by the sum of incomes of all individual residents at home, including wages, pensions, and any other income at the time of the collection. The number of rooms and the number of residents per domicile were collected using a direct question.

### 2.9 COVID-19 antibodies

Immunoglobulin M (IgM) and Immunoglobulin G (IgG) analysis for SARS CoV-2 were performed through the chemiluminescence test (Abott) in serum samples of the participants, following the manufacturer’s protocol.

### 2.10 Data analysis

Exploratory data analysis was performed by calculating means (standard deviations) and frequency (percentages) of the variables of interest (overall and stratified by tertiles of total PA levels). The variables associated with total PA levels (as a continuous variable) were identified using exploratory generalized linear regression models with a logarithmic link and gamma distribution due to the asymmetric and heteroscedastic nature of the residuals. The coefficients (beta) were exponentiated (Exp β) to facilitate the interpretation of the results. Only variables with *p* ≤ 0.10 in the univariate analysis were included in the multivariate model. A sensitivity analysis including only the variables with *p* ≤ 0.05 in the univariate analysis in the full multivariate model was also performed. The backwards method was used to sequentially remove variables with *p* > 0.05 values in multivariate analysis, until the final model maintained only those with *p* ≤ 0.05.

The Research Electronic Data Capture (REDCap) web application was used for data management and the data analysis was conducted using Stata 13.0 software. Statistical significance was set at *p* ≤ 0.05 for all analyses.

## 3 Results

Of 350 invited patients, 187 agreed to participate in the study and were included in the analysis. The percentage of individuals who met the 2020 WHO PA recommendation was 52.0%. The general characteristics of the patients included in the study are presented in Table 1 (overall and stratified by tertiles of PA). The mean age of individuals included in the study was 61.1 years, with 62.0% of women. The mixed race was predominant (50.8%), with most patients having <9 years of schooling (69.5%). Overall, those in the highest tertile of PA were younger, less likely to present comorbidities (arterial hypertension, diabetes mellitus, and dyslipidemia), and with a lower percentage of severe cardiac forms of ChD.

**Table 1:** Characteristics of participants included in the study (*n* = 187).

Variables	General	Tertiles of total PA (MET.min^−1^. week ^1^)			*p*-value for trend
	*n* = 187	1° tertile (*n* = 63; 513.3)	2° tertile (*n* = 62; 2025.7)	3° tertile (*n* = 62; 5378.9)	
Age (years)	61.1 (± 11.6)	64.2 (± 11.3)	59.6 (± 10.3)	59.3 (± 12.6)	0.02
Sex (%)					
Women	62.0% (116)	60.3% (38)	56.5% (35)	69.4% (43)	0.30
Men	38.9% (71)	39.7% (25)	43.5% (27)	30.6% (19)	
Race (%)					
White	23.0% (43)	28.6% (18)	25.8% (16)	14.5% (9)	0.09
Black	29.8% (37)	15.9% (10)	24.2% (15)	19.4% (12)	
Mixed	50.8% (95)	52.4% (33)	45.2% (28)	54.8% (34)	
Others	6.4% (12)	3.2% (2)	4.8% (3)	11.3% (7)	
Marital status					
Married/Stable union	52.9% (99)	49.2% (31)	61.3% (38)	48.4% (30)	0.93
Others	47.1% (88)	50.8% (32)	38.7% (24)	51.6% (32)	
Schooling (years)					
< 9	69.5% (130)	69.8% (44)	70.9% (44)	67.7% (42)	0.69
≥ 9–12	13.9% (26)	9.5% (6)	17.7% (11)	14.5% (9)	
≥ 12	16.6% (31)	20.6% (13)	11.3% (7)	17.7% (11)	
Number of rooms per domicile	4.63 (±1.40)	4.73 (±1.62)	4.63 (±1.32)	4.52 (±1.22)	0.39
Number of residents per domicile	1.71 (±1.53)	1.86 (±1.59)	1.63 (±1.43)	1.65 (±1.57)	0.44
Income per capita*					
Up to 1 minimum wage	54.0% (101)	66.7% (42)	45.2% (28)	50.0% (31)	0.06
Above 1 minimum wage	45.9% (86)	33.3% (21)	54.8% (34)	50.0% (31)	
BMI (kg/m^2^)	28.2 (± 5.11)	28.9 (± 5.1)	27.2 (± 4.8)	28.4 (± 5.3)	0.57
Indeterminate form	32.6% (61)	25.4% (16)	35.5% (22)	37.1% (23)	0.16
Cardiac form	63.6% (119)	71.4% (45)	61.3% (38)	58.1% (36)	0.12
Stage cardiac form					
Without CCC	36.4% (68)	28.6% (18)	38.7% (24)	41.9% (26)	0.03
A	26.2% (49)	20.6% (13)	22.6% (14)	35.5% (22)	
B1	20.3% (38)	30.2% (19)	16.1% (10)	14.5% (9)	
B2	5.4% (10)	4.8% (3)	11.3% (7)	0% (0)	
C	11.8% (22)	15.9% (10)	11.3% (7)	8.1% (5)	
Digestive form	13.4% (25)	14.3% (9)	11.3% (7)	14.5% (9)	0.97
COVID-19 antibodies					
IgM					
Positive	11.2% (21)	11.1% (7)	9.7% (6)	12.9% (8)	0.75
Negative	88.8% (166)	88.9% (56)	90.3% (56)	87.1% (54)	
IgG					
Positive	18.7% (35)	15.9% (10)	16.1% (10)	24.2% (15)	0.24
Negative	81.3% (152)	84.1% (53)	83.9% (52)	75.8% (47)	
Comorbidities					
Systemic arterial hypertension	70.0% (131)	85.7% (54)	62.9% (39)	61.3% (38)	0.003
Diabetes mellitus	21.4% (40)	28.6% (18)	22.6% (14)	12.9% (8)	0.04
Dyslipidemia	51.9% (97)	61.9% (39)	50.0% (31)	43.6% (27)	0.04
Obesity	29.9% (56)	30.2% (19)	29.0% (18)	30.6% (19)	0.95
Respiratory disease	14.9% (28)	9.5% (6)	12.9% (8)	22.6% (14)	0.05
Non-chagasic heart disease	3.2% (6)	1.6% (1)	3.2% (2)	4.8% (3)	0.31
Other chronic diseases	14.9% (28)	15.9% (10)	16.1% (10)	12.9% (8)	0.64
Self-reported anxiety during quarantine	47.1% (88)	42.9% (27)	48.4% (30)	50.0% (31)	0.42
Self-reported fear during quarantine	51.3% (96)	53.9% (34)	51.6% (32)	48.4% (30)	0.53
Self-reported sadness during quarantine	56.2% (105)	55.6% (35)	54.8% (34)	58.1% (36)	0.78
Self-reported depression during quarantine	26.2% (49)	30.2% (19)	30.6% (19)	17.7% (11)	0.12

Race: others (yellow and indigenous); Marital status: others [single, divorced, widower (a)]; BMI, body mass index; No CCC, without chronic chagasic heart disease; IgM, Immunoglobulin M; IgG, Immunoglobulin G; Other chronic diseases: cancer, kidney disease, liver disease, another chronic disease; METs, Metabolic equivalent of the task.

**Figure 1:**
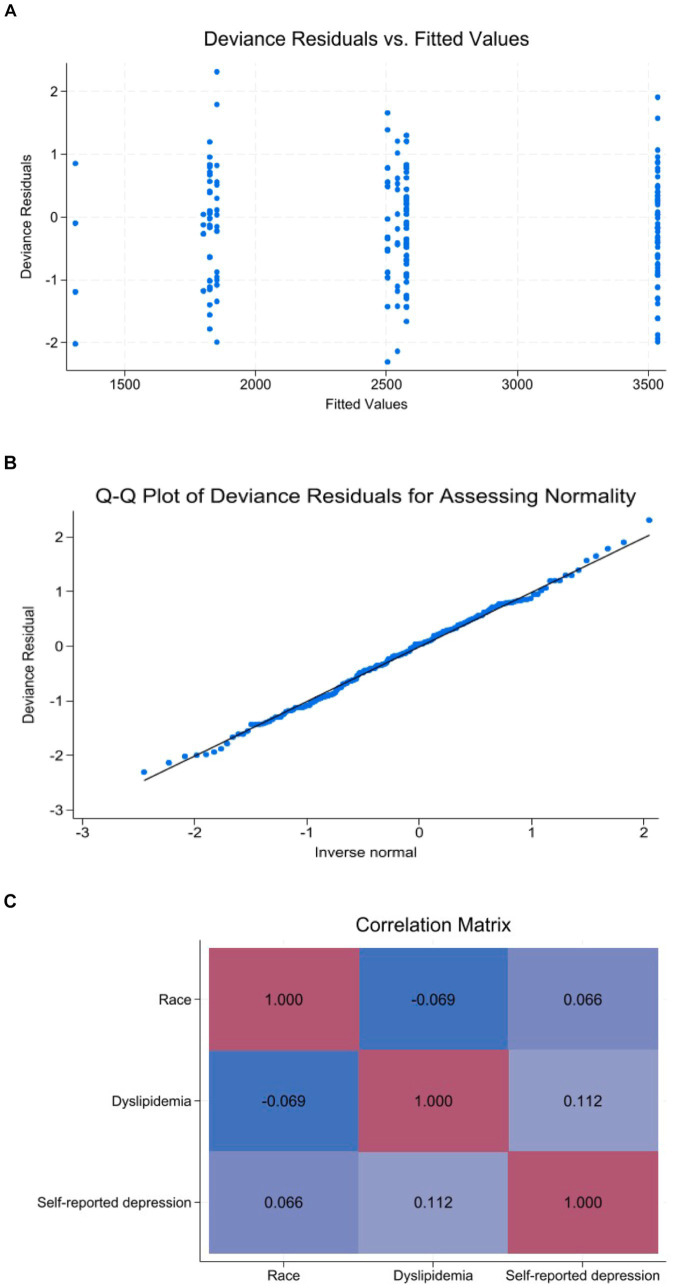
Diagnostic plots for demonstration of GLM assumptions. **(A)** Deviance residuals vs. fitted values. **(B)** Q-Q plot of deviance residuals. **(C)** Correlation matrix map.

Table 2 depicts the univariate association between investigated exposure variables and total PA levels. The variables that were statistically significant associated with total PA levels were age (Exp β = 0.99; 95% CI 0.97 to 0.99), non-white race (Exp β = 1.37; 95% CI 0.99 to 1.89), education levels ≥12 years (Exp β = 1.41; 95% CI 1.00 to 1.99), income *per capita* above 1 minimum wage (Exp β = 1.31; 95% CI 1.00 to 1.70), diabetes (Exp β = 1.53; 95% CI 1.12 to 2.11), dyslipidemia (Exp β = 0.69; 95% CI 0.53 to 0.89) and self-reported depression during quarantine (Exp β = 0.71; 95% CI 0.53 to 0.96). In the multivariate model, the variables that were independently associated with total PA levels were non-white race (Exp β = 1.39; 95% CI 1.02 to 1.90), dyslipidemia (Exp β = 0.73; 95% CI 0.56 to 0.95) and self-reported depression during quarantine (Exp β = 0.71; 95% CI 0.52 to 0.96) (Table 3). These variables remained consistent in the sensitivity analysis, which included only the variables with *p* ≤ 0.05 in the univariate analysis in the full multivariate model. Analysis of deviance residuals and examination of the correlation between explanatory variables did not demonstrate deviations from GLM assumptions (Figure 1).

**Table 2:** Univariate linear regression for association between exposure variables and total PA (METs) in patients with Chagas disease (*n* = 187).

Variables	Exponential	95%CI	*p*-value
Age (years)	0.99	0.97 to 0.99	0.01
Sex (%)			
Women	Reference		
Men	1.01	0.77 to 1.34	0.92
Race (%)			
White	Reference		
Non-white	1.37	0.99 to 1.89	0.05
Marital status			
Married/stable union	Reference		
Single/divorced/widowed	0.92	0.70 to 1.20	0.54
Schooling (years)			
<9	Reference		
≥9–12	1.17	0.80 to 1.69	0.42
≥ 12	1.41	1.00 to 1.99	0.05
Number of rooms per domicile	0.96	0.88 to 1.09	0.65
Number of residents per domicile	1.00	0.92 to 1.09	0.91
Income per capita *			
Up to 1 minimum wage	Reference		
Above 1 minimum wage	1.31	1.00 to 1.70	0.04
BMI (kg/m^2^)	0.99	0.97 to 1.02	0.96
Indeterminate form	1.29	0.97 to 1.73	0.08
Stage cardiac form			
Without CCC	Reference		
Cardiac without HF	0.81	0.61 to 1.09	0.16
Cardiac with HF	0.71	0.45 to 1.12	0.14
Digestive form	0.91	0.62 to 1.35	0.65
COVID-19 antibodies			
IgM			
Positive	0.82	0.54 to 1.24	0.34
IgG			
Positive	0.96	0.68 to 1.36	0.83
Comorbidities			
Systemic arterial hypertension	1.29	0.96 to 1.75	0.09
Diabetes mellitus	1.53	1.12 to 2.11	0.008
Dyslipidemia	0.69	0.53 to 0.89	0.006
Obesity	1.00	0.75 to 1.34	0.98
Respiratory disease	1.29	0.88 to 1.88	0.19
Non-chagasic heart disease	0.86	0.40 to 1.83	0.68
Other chronic diseases	0.87	0.59 to 1.27	0.47
Self-reported anxiety during quarantine	0.97	0.74 to 1.26	0.80
Self-reported fear during quarantine	0.89	0.68 to 1.16	0.38
Self-reported sadness during quarantine	0.93	0.71 to 1.22	0.61
Self-reported depression during quarantine	0.71	0.53 to 0.96	0.03

Race: no white (black, mixed, others: yellow and indigenous); Marital status: others [single, divorced, widower (a)]; BMI, body mass index; CCC, chronic chagasic heart disease; HF, heart failure; IgM, Immunoglobulin M; IgG, Immunoglobulin G; Other chronic diseases: cancer, kidney disease, liver disease, another chronic disease; METs: Metabolic equivalent of the task.

**Table 3:** Multivariate linear regression for association between exposure variables and total PA (METs) in patients with Chagas disease (*n* = 187).

Variables	Exponential	95%CI	*p*-value
Race (%)			
White	Reference		
Non-white	1.39	1.02 to 1.90	0.04
Dyslipidemia	0.73	0.56 to 0.95	0.02
Self-reported depression during quarantine	0.71	0.52 to 0.96	0.03

Race: no white (black, mixed, others: yellow and indigenous). Model Fit Log likelihood: −1,653.11; BIC: -810.75; AIC: 17.72.

## 4 Discussion

The present study revealed a relatively low adherence (52%) to the 2020 WHO PA recommendations among a sample of urban patients with ChD during the COVID-19 pandemic. Additionally, three variables—race, presence of dyslipidemia, and self-reported depression—were independently associated with overall levels of PA.

Studies assessing PA levels in the general population during the COVID-19 pandemic have reported conflicting estimates (19–21). For example, a cross-sectional study in Brazil, which online surveyed 1,853 individuals of both sexes, noted a decline in the proportion of physically active individuals during the COVID-19 pandemic compared to the pre-pandemic period (76.5% vs. 90.5%, respectively) (12). In contrast, another cross-sectional study with 771 students from a Brazilian public university, using an online questionnaire, found that 50.2% were classified as physically active during the COVID-19 pandemic (22), a lower percentage in comparison to the previously mentioned study. Further supporting the decreased levels of PA during the COVID-19 pandemic, a Canadian online survey involving 1,098 adults reported an even lower prevalence (36.6%) of physically active individuals during that period (23), with 40.5% of the physically inactive participants reporting that became less active after the implementation of pandemic restrictions recommendations, underscoring the detrimental impact of social isolation on overall PA levels.

The existing literature on the PA levels of individuals with ChD is limited, particularly in the context of the COVID-19 pandemic. In a prior cross-sectional observational study conducted by our research group between 2014 and 2017, encompassing 361 individuals with ChD under the care of the same institution, 74.2% were identified as physically active based on the same WHO’s PA definition (24). Therefore, considering that participants were selected from the same population base and presented similar clinical and demographic characteristics, a decrease in PA levels was observed when comparing data before and after the COVID-19 pandemic period, which may be attributed to the social isolation measures adopted during this period (19).

Three variables were independently associated with the total PA: race, dyslipidemia, and self-reported depression during quarantine. In our study, non-white race was associated with an increased total PA level (25, 26). In Brazil, non-white individuals often occupy blue-collar occupations due to their lower socioeconomic status. These occupations, characterized by physically demanding tasks, may contribute to elevated levels of total PA. Throughout the COVID-19 pandemic, many of these individuals lacked the financial resources to stay home, as their livelihoods depended on continued work, and they were unable to engage in remote work. In some cases, there was even an increase in workload during the pandemic period. These factors may explain the observed higher levels of PA levels observed in the non-white participants in the present study (27, 28).

In our study, the presence of dyslipidemia was significantly associated with decreased PA levels. Individuals with dyslipidemia usually present inadequate health habits, such as an unhealthy diet and low levels of PA (29). Since dyslipidemia is often a silent condition that is frequently underestimated, it is possible to speculate that individuals with dyslipidemia may have further decreased their levels of PA during the pandemic due to their usual poor lifestyle habits. However, due to the cross-sectional design of the present study, the direction of this association is difficult to establish, with most of previous examining the inverse direction, suggesting that reduced PA levels were associated with a higher risk of dyslipidemia (30–32).

Finally, our study showed that self-reported depression during quarantine was independently associated with decreased total PA levels. The COVID-19 pandemic has exacerbated mental health challenges (33–35). A major contributor to this scenario has been social distancing, a successful measure in controlling the transmission of COVID-19, but with several negative repercussions on people’s well-being due to the loss of social support and increased feelings of loneliness and isolation (8, 10). A cross-sectional study carried out in Colombia with 431 individuals assessing the effects of the COVID-19 lockdown on PA levels and mental health found a substantial negative compromise of the psychological well-being (36). Moreover, another cross-sectional study conducted in Spain with 483 individuals found that individuals who complied with the WHO’s 2020 PA recommendations showed greater resilience, positive affect, and fewer depressive symptoms that may have positively contributed to situations of isolation (21).

The present study has some limitations. The cross-sectional design of the study prevents us from drawing conclusions about the causal relationship between the investigated variables with total PA levels. Our sample was composed of patients regularly followed-up in a national reference center, which may limit external validity. Moreover, self-reported measurements of mental health aspects (anxiety, depression, fear, and sadness) may have suffered some influence from motivational, cultural, and social factors, such as the low education levels of the studied sample. On the other hand, this is the first study that evaluated the level of PA during the COVID-19 pandemic in patients with ChD.

In conclusion, we found a relatively low compliance with the WHO PA recommendations during the COVID-19 pandemic period in a sample of patients with ChD. Non-white race was positively associated with total levels of PA, while dyslipidemia, and self-reported depression during quarantine were negatively associated with total levels of PA. In this scenario, identifying patient characteristics associated with lower PA levels may facilitate the development of tailored approaches to encourage more ChD patients to become physically active.

## Data availability statement

The raw data supporting the conclusions of this article will be made available by the authors upon reasonable request.

## Ethics statement

The studies involving humans were approved by Research Ethics Committee of the Evandro Chagas National Institute of Infectious Diseases. The studies were conducted in accordance with the local legislation and institutional requirements. The participants provided their written informed consent to participate in this study.

## Author contributions

IX: Data curation, Writing – original draft, Writing – review & editing. PA: Writing – review & editing. RV: Writing – review & editing. TB: Writing – review & editing. LP: Writing – review & editing. MH: Writing – review & editing. LS: Writing – review & editing. GS: Methodology, Writing – review & editing. FM-R: Writing – review & editing. FM: Methodology, Writing – review & editing. AC: Writing – review & editing. MQ: Formal analysis, Conceptualization, Writing – review & editing. AH-M: Methodology, Writing – review & editing. IA: Writing – review & editing. AJ: Methodology, Writing – review & editing. RP: Writing – review & editing. IG: Writing – review & editing. VP: Writing – review & editing. TG: Writing – review & editing. RS: Methodology, Supervision, Writing – review & editing, Funding acquisition. MM: Writing – review & editing, Methodology, Supervision, Writing – original draft.
